# Impact of switching from polymerase chain reaction-only to two-step *Clostridioides difficile* testing in a large hospital system

**DOI:** 10.1017/ash.2025.10230

**Published:** 2025-11-24

**Authors:** Sapana R. Gupta, Tyler M. Selig, Kathryn Evey, Michael Rossi, Adam M. Burton, Curtis Petruzzelli, Jacqueline J. Chu, Wen Ting Yang, James Scharfen, Joshua Ray Tanzer, John R. Lonks, Colleen R. Kelly

**Affiliations:** 1 Department of Medicine, Brown University Health, Providence, RI, USA; 2 Division of Gastroenterology, Stony Brook University Hospital, Stony Brook, NY, USA; 3 Division of Infectious Disease, Warren Alpert Medical School of Brown University, Providence, RI, USA; 4 Division of Gastroenterology, Icahn School of Medicine at Mount Sinai Morningside/West, New York, NY, USA; 5 Division of Gastroenterology, Hepatology, and Endoscopy, Brigham and Women’s Hospital, Boston, MA, USA

## Abstract

**Introduction:**

*Clostridioides difficile* is the primary cause of healthcare-associated infectious diarrhea in hospitalized patients. The most common laboratory testing methods for *C. difficile* infection (CDI) are toxin detection via enzyme immunoassay (EIA) and polymerase chain reaction (PCR), which detect a toxogenic strain. This study examines the impact of Rhode Island’s largest hospital system changing from PCR-only to two-step CDI testing.

**Methods:**

A retrospective cohort study of 2,173 adult inpatients was conducted. Patients were grouped into two cohorts: those tested for toxigenic *C. difficile* via PCR-only (June 2019–May 2021, *n* = 1,194) and those tested with the two-step algorithm (June 2021–May 2023, *n* = 979). Cluster analysis identified patient risk groups for hypothesis generation, and complications such as death, colectomy, intensive care unit ICU transfer, and 30-day readmission were compared across these groups.

**Results:**

In the moderate-risk group, there was a significant reduction in ICU transfers and readmission rates with the two-step testing by 5% and 7%, respectively. There were no other significant differences in complications between testing groups. Anti-CDI antibiotics were discontinued in 15% (*n* = 106) of EIA-negative patients in the two-step testing group. Moderate-risk patients were less likely to have treatment discontinued than severe-risk patients (OR = 2.00, *p* = 0.016).

**Discussion:**

The two-step testing algorithm did not negatively affect patient outcomes and led to a modest decrease in anti-CDI treatment, supporting the safety of two-step CDI testing in hospitalized patients.

## Introduction

*Clostridioides difficile* is the most common microbial pathogen causing nosocomial infections.^1^ Thus, diagnosing *C. difficile* infections (CDIs) in the most efficacious and cost-conscious way is important for any hospital system. Diagnosis of CDI requires the acute onset of diarrhea, greater than or equal to three episodes of watery stool in 24 hours, and confirmatory stool testing.^2^ Three frequently used laboratory methods of diagnosing CDI include the enzyme immunoassay (EIA) for glutamate dehydrogenase (GDH), EIA for free toxin A and B, and nucleic acid amplification testing (NAAT) polymerase chain reaction (PCR) testing. The GDH EIA assesses the stool for the presence of GDH, an enzyme produced by both the toxigenic and non-toxigenic *C. difficile* strains, which is a sensitive but not specific test for CDI.^3^ The toxin EIA for *C. difficile* detects the presence of free toxins A and B in the stool, with high specificity and low sensitivity for CDI, which may lead to underdiagnosis. Highly sensitive NAAT, such as PCR, tests for the presence of a toxigenic strain of the organism but cannot differentiate between active infection and colonization. PCR testing can lead to overdiagnosis and overtreatment. Additionally, it can inadvertently increase the rate of hospital-onset CDI.

In 2017, the National Healthcare Safety Network implemented guidelines for CDI surveillance using a multistep testing algorithm, wherein the last diagnostic result within a testing case determines reportable CDI events.^4^ This surveillance change, together with concerns about inflated hospital-onset CDI rates associated with PCR-only testing, prompted many institutions to adopt multi-step diagnostic algorithms in an attempt to reduce the over-diagnosis of CDI using standalone PCR testing. These typically pair an initial PCR test with reflex toxin EIA to improve specificity and align with national reporting practices.

Discontinuation of therapy in patients with *C. difficile* PCR-positive and toxin EIA-negative can lead to adverse clinical outcomes. For example, a patient presented to one of our hospitals with watery diarrhea and imaging consistent with pancolitis; she was started on empiric CDI therapy. However, stool testing was PCR-positive/EIA-negative and treatment was subsequently discontinued. The patient was discharged but readmitted ten days later with recurrent diarrhea, at which time stool testing was both PCR-positive/EIA-positive. Despite resumption of oral vancomycin and addition of intravenous metronidazole, her clinical status and renal function deteriorated, and her WBC increased from 11,100/mm^3^ to 27,600/mm^3^. She ultimately underwent fecal microbiota transplantation on day 10 of hospitalization, resulting in clinical improvement. This case highlights that withholding treatment in PCR-positive/EIA-negative patients may contribute to significant morbidity.

In June 2021, Rhode Island’s largest hospital system changed from using PCR-only testing to a two-step process, starting with PCR and, when positive, reflexing to toxin EIA confirmatory testing. The primary objective of this study was to evaluate the impact of implementing a two-step CDI testing algorithm on clinical outcomes as assessed by rates of ICU transfer, hospital readmission, and mortality between the NAAT-only and two-step testing periods. The secondary objective was to examine CDI-targeted antibiotic use, including initiation, continuation, and discontinuation, and assess whether outcomes varied across patient subgroups.

## Methods

### Study design and subjects

A retrospective chart review of 2,173 adult inpatients, who had positive testing for CDI at either of two tertiary care hospitals between June 2019 and May 2023, was conducted. Patients were divided into two groups: those tested for toxigenic *C. difficile* via PCR-only between June 2019 and May 2021 (*n* = 1,194) and those using the two-step test between June 2021 and May 2023 (*n* = 979). During both the PCR-only and two-step testing periods, PCR testing for *C. difficile* was performed locally at each hospital using the Cepheid GeneXpert platform on demand, with results available the same day the specimen was received by the laboratory. During the two-step period, EIA testing for toxin was performed using the ImmunoCard Toxins A&B assay (Meridian Bioscience); testing was batched and performed once daily; hence, results were available the same day or the following day. For PCR-positive results, reports stated “See EIA results for further information,” and for PCR+/EIA- results, the comment noted that a negative toxin may reflect colonization and should be interpreted in clinical context. Patients were excluded from the study if they were less than 18 years of age. This study was approved by the Lifespan Institutional Review Board.

### Data collection

The electronic medical record, Epic, was reviewed using firewall-secure computers and stored data on the password-protected Research Electronic Data Capture database. The data collected included: sex, age, race/ethnicity, diarrhea reported on admission, 3 or more episodes of diarrhea in 24 hours, laxative exposure at the time of CDI testing, CDI test results (NAAT, EIA) for each occasion tested, date of CDI diagnosis, the name of the anti-CDI therapies used (vancomycin, fidaxomicin, IV metronidazole, other, or none), whether anti-CDI therapies were stopped versus continued after negative EIA, highest white blood cell count (WBC) and serum creatinine within 3 days pre/postCDI testing, abdominal CT scan results within 7 days pre/postCDI testing, colonoscopy or sigmoidoscopy reports during the same hospitalization, colectomy during hospitalization, death during hospitalization, readmission within 30 days, intensive care unit (ICU) admission during hospitalization, and ICU transfer during hospitalization.

### Statistical analysis

To account for potential selection bias related to patient severity, we conducted a cluster analysis using clinical and laboratory data available at presentation. Clinical severity groups were based on demographics (gender and age) and clinical presentation (diarrhea at admission, maximum WBC, and maximum creatinine). This approach grouped patients with similar illness severity to minimize confounding between disease presentation and provider decision-making. This data-driven approach also mitigated the need for prespecifying individual covariates, which could be influenced by multicollinearity among laboratory variables. Logistic regression models were used to assess associations between testing and treatment strategy and three prespecified outcomes: ICU transfer during hospitalization, readmission, and mortality. Change over time was modeled as a linear function in terms of days.

The number of clusters was determined through examination of the dendrogram and agglomeration coefficients, which suggested a range of 2–5 potential groups. Based on these assessments, a three-cluster solution was selected as the most clinically informative.

The difference in estimates between the preperiod and the postperiod was tested on the T-distribution within the model. *P* value < .05 was considered statistically significant. The null hypothesis was no difference between the two periods of testing.

## Results

A three-group solution was selected from the cluster analysis, based on gender, age, diarrhea at admission, WBC, and creatinine (Table 1). Following implementation of the two-step testing algorithm, the rate of National Healthcare Safety Network-defined hospital-onset CDI decreased markedly, from 5.7 to 1.3 per 10,000 patient days at Hospital A and from 6.7 to 1.5 per 10,000 patient days at Hospital B. Early in the transition period, occasional PCR-positive samples were not reflexed to EIA because of workflow adjustments. The clinical characteristics, changes to CDI treatments, and health outcomes are discussed individually.

**Table 1.** Comparison of demographics, inpatient ICU transfers, hospital readmission, and mortality of CDI groups

	Group 1-mild CDI	Group 2- moderate CDI	Group 3-severe CDI
**Demographics**			
Sample size, n	688	1 140	345
Female gender, %	49	58	39
Mean age, years	41	73	66
Diarrhea at admission, %	53	48	45
WBC maximum (× 10^9^/L)	8.8	15.3	16.3
Creatinine maximum (mg/dL)	0.9	1.2	5.7
Treatment discontinuation, %	16	11	21
**ICU transfer during stay, n/N (%)**			
PCR-Only	17/317 (5%)	39/482 (8%)	27/149 (18%)
NAAT+, EIA not performed	2/79 (3%)	5/157 (3%)^a^	13/60 (22%)
NAAT +, EIA -, treatment cntinued	4/177 (2%)	27/347 (8%)	11/89 (12%)
NAAT +, EIA -, treatment discontinued	1/34 (3%)	4/45 (9%)	1/23 (4%)
**Readmission, n/N (%)**			
PCR-Only	87/316 (28%)	123/472 (26%)	46/145 (32%)
NAAT+, EIA not performed	13/79 (16%)	35/157 (22%)	14/60 (23%)
NAAT +, EIA -, treatment continued	55/177 (31%)	66/345 (19%)^a^	33/88 (38%)
NAAT +, EIA -, treatment discontinued	12/34 (35%)	14/45 (31%)	7/23 (30%)
**Mortality, n/N (%)**			
PCR-Only	10/320 (3%)^b^	24/483 (5%)	22/150 (15%)
NAAT+, EIA not performed	1/79 (1%)^b^	6/156 (4%)	9/60 (15%)
NAAT +, EIA -, treatment continued	1/178 (1%)^b^	23/347 (7%)	9/89 (10%)
NAAT +, EIA -, treatment discontinued	0/34 (0%)^b^	3/45 (7%)	3/23 (13%)

a *P* < 0.05 when comparing to PCR-only percentage.

b Group 1 was excluded from the analysis of mortality because there was only one death for NAAT + EIA not performed, one death for NAAT + EIA - treatment continued, and no deaths for NAAT +, EIA -, treatment discontinued. Including these groups in the analysis would have made inferences and estimates unreliable.

### Group 1: mild CDI

This group was composed of 688 patients, 317 patients in the PCR-only period and 290 patients during the two-step period. 49% of the total number of patients were female (95% CI [45, 52]) with a mean age of 41 years (95% CI [40, 43]). Slightly over half (53%, 95% CI [49, 56]) of patients had diarrhea upon admission. Maximum WBC was 8.8 × 10^9^/L (95% CI [8.3, 9.1]) and maximum creatinine level was .9 mg/dL (95% CI [.8, .9]).

There were 34/211 (16%) patients who had anti-CDI therapies stopped after negative EIA. During the PCR-only period, 17/317 (5%) patients required an ICU transfer during their admission. While during the two-step testing period, regardless of test result or treatment continuation, all patients had slightly lower rates of ICU transfer, though no differences were significant. 87/316 (28%) patients in the PCR-only group were readmitted to the hospital. There was a slight, but not statistically significant (*P* > .05), increase in readmission during the two-step period for patients who were NAAT positive, EIA negative, and had treatment discontinued (12/34, 35%). No significant differences were found for any of the other patient treatment groups (NAAT+, EIA not performed, 13/79 16%; NAAT+, EIA-, treatment continued, 55/177, 31%). The mortality during the PCR-only period was 10/320 (3%). Mortality rates decreased across test results, though the infrequency precluded testing the magnitude of difference.

### Group 2: moderate CDI

This group was composed of 1,140 patients, 482 patients in the PCR-only period and 549 patients during the two-step period. 58% were female (95% CI [56, 61]) and had a mean age of 73 years (95% CI [73, 74]). About half (48%, 95% CI [45, 51]) of patients had diarrhea upon admission. Maximum WBC was 15.3 × 10^9^/L (95% CI [14.8, 15.8]) and maximum creatinine level was 1.2 mg/dL (95% CI [1.2, 1.2]).

There were 45/392 (11%) patients who had anti-CDI treatments stopped after positive PCR and negative EIA. There was a significant decrease in the ICU transfer rate for patients who were NAAT positive, but EIA was not performed, 5/157 (3%, *p* < .05), suggesting providers may have been more selective in the patients for whom they continued or discontinued treatment. ICU transfer rates were similar among patients who were NAAT positive and EIA negative, regardless of treatment continuation (treatment continued, 27/347, 8%; treatment discontinued, 4/45, 9%). Among patients in the two-step period who were NAAT positive, EIA negative, and treatment was continued, 66/345 (19%) were readmitted, a significant decrease (*p* < .05). Changes were not significantly different for patients for whom EIA was not performed (35/157, 22%) or for whom treatment was discontinued (14/45, 31%). The mortality rate during PCR-only testing was 24/483 (5%). Changes in mortality rates were not significantly different for any two-step test result group.

### Group 3: severe CDI

This group was composed of 345 patients, 149 during the PCR-only period and 172 during the two-step period. 39% were female (95% CI [35,44]) and had a mean age of 66 years (95% CI [65,68]). Less than half of the patients (45%, 95% CI [40, 50]) had diarrhea upon admission. Maximum WBC was 16.3 × 10^9^/L (95% CI [15, 17.7]) and maximum creatinine level was 5.7 mg/dL (95% CI [5.4, 6]).

Within this group, 23/112 (21%) had anti-CDI treatments stopped after negative EIA. There were a range of increases and decreases in ICU transfer rates in the two-step period, though none were significantly different from the PCR-only period (NAAT+, EIA not performed, 13/60, 22%; NAAT+, EIA-, treatment continued, 11/89, 12%; NAAT+, EIA-, treatment discontinued, 1/23, 4%). As with ICU transfer, the rates of readmission were not meaningfully different in the two-step testing period. The mortality rate during the PCR-only period was 22/150 (15%). The mortality rates during the two-step testing period were similar or slightly lower, but not significantly different.

### Post hoc sensitivity and power analysis

The primary concern was that there would be an increase in moderate to severe adverse outcomes when switching from PCR-only to two-step testing, specifically for patients who were NAAT-positive but EIA-negative when CDI treatment was discontinued. Patients in this group received the recommended treatment, but if their clinical outcomes deteriorated it would provide evidence that patients with false-negative EIA tests are going untreated. When stratifying the analysis by cluster-identified groups, some of the frequencies of each outcome event were low. A sensitivity analysis was performed, collapsing across cluster-identified groups, directly comparing solely based on treatment received regardless of underlying health condition. None of the comparisons from PCR-only to two-step testing were significant (*p* > .05).

Additionally, a post hoc power analysis was performed, to further investigate how the sample size may have affected results. The magnitude of difference that would have 80% likelihood of being detected against a null hypothesis of no difference with a 5% two-tailed type one error rate was calculated for each outcome. The preperiod probability of each outcome was assumed to be the true probability, to derive what magnitude of increase would need to be demonstrated among patients who were NAAT-positive and EIA-negative with treatment discontinued, to be significant. The rate ratio required to have sufficient power ranged from 1.5 to 2.4, and the odds ratio required ranged from 1.8 to 2.6. Importantly, per recommendations by Cohen, these are not generally viewed as large differences, supporting that the analysis was powered to detect small to moderate changes from PCR-only to two-step testing.^5^ While researchers must always be cautious in accepting the null hypothesis of no difference, the posthoc power analysis supports that there is inadequate evidence that the two-step testing protocol adversely affected patient outcomes.

## Discussion

The aim of this study was to determine if two-step (NAAT reflexing to EIA) testing for CDI, resulted in adverse clinical outcomes or improvements in antibiotic stewardship. We did not find evidence of moderate to severe adverse patient outcomes after the change from PCR-only to two-step testing, providing evidence that the two-step testing protocol was not a risk to patient safety. The change in testing was associated with anti-CDI antibiotic discontinuation in some patients, resulting in both treatment cost and antibiotic stewardship benefits for the patients and the healthcare system.

Our study adds to previous literature examining the utility of two-step testing for CDI diagnosis. Polage et al found that PCR-positive, toxin-negative patients had milder symptoms, fewer complications, and significantly lower mortality compared to PCR-positive, toxin-positive patients, with outcomes similar to those without CDI. This suggests that relying solely on molecular tests can lead to overdiagnosis, overtreatment, and underscoring the need to include toxin testing in CDI diagnosis.^6^ In addition, a systematic review and meta-analysis of over 12,000 patients assessed outcomes in CDI cases that were NAAT-positive but toxin-negative. They found that while treatment in this group may reduce 30-day mortality, it does not significantly affect in 60-day recurrence, emphasizing the need for individualized decisions for CDI treatment.^7^ Hogan et al. conducted a quasi-experimental, non-inferiority study at a single-center, tertiary academic hospital to compare clinical outcomes in PCR-positive, toxin-negative patients. The study assessed outcomes under two reporting strategies: PCR-only reporting (which led to most patients being treated) versus toxin-only reporting (resulting in most patients remaining untreated). This study did not show increased lack of diarrhea resolution or 30 day all-cause mortality rates in the untreated group, reaffirming the importance of toxin-based testing.^8^ Like these prior studies, we found no evidence of moderate to severe adverse patient outcomes when switching from PCR-only to two-step testing.

Implementation of two-step testing can provide financial benefits for patients and the healthcare system. The substantial cost difference between fidaxomicin ($4,300) and vancomycin ($75) for a 10-day course underscores the importance of diagnostic stewardship and judicious treatment selection to optimize both clinical and economic outcomes.^9^ Furthermore, as a component of the Centers for Medicare & Medicaid’s Hospital-Acquired Condition Reduction Program, lower rates of CDI are linked to increased budgetary savings.^10^ Thus, there is a fiscal incentive for healthcare systems to prevent and accurately diagnose CDI and other hospital-acquired infections. In addition, treating only EIA-positive patients prevents unnecessary antibiotic exposure and perpetuation of gut dysbiosis, decreasing the risk of vancomycin-resistant enterococci.

Interestingly, the rates for discontinuation of CDI therapy differed by severity of presentation. In this study, 15% of patients overall with discordant testing had treatment discontinued. The change in testing was made without actively educating clinicians on result interpretation. Therefore, providers may have been erring on the side of caution when deciding to treat. In this sample of patients, those with severe disease had anti-CDI antibiotics discontinued at a higher rate than those considered to have moderate disease. The discrepancy may be attributed to diarrhea due to an alternate diagnosis and incidental colonization with *C. difficile* or transition to hospice-level of care due to severe illness. Parameters of patients in the moderate CDI risk group (majority female, older age, leukocytosis) may be most representative of true CDI and thus were more likely to be treated and benefit from antibiotic therapy. Therefore, increased education regarding the high accuracy of the two-step testing may result in greater improvements in antibiotic stewardship in the future.

The strengths of this study include its large sample size (>2,000 patients) and long duration of review (4 yr). However, there are several limitations to this study. One limitation is the lack of baseline creatinine levels to allow for identification of patients who experienced an acute change in kidney function in the setting of CDI, versus those with stable chronic kidney disease. Another drawback to the study was that the subjects were from one center in the Northeastern United States, which may limit its generalizability. Lastly, the PCR-only and two-step testing groups are sequential and not contemporaneous, which may reflect some confounding changes in the approach to CDI treatment. Future research will be necessary to identify which patients with discordant testing can have treatment deferred and which patients should still receive treatment as well as recurrence-related metrics, such as downstream CDI testing or positive retesting. In addition, stratifying patients with preexisting chronic kidney disease will be helpful to better assess the true effect of CDI on kidney function.

## Conclusion

Switching from PCR-only to two-step testing for CDI did not result in worse clinical outcomes, reinforcing its safety. While no differences in ICU transfer or readmission were observed among untreated PCR+/EIA- patients, we acknowledge that other adverse events may have occurred but were not captured in this analysis, representing a limitation of the study. Additionally, the change improved antibiotic stewardship, underscoring its value for both patient care and healthcare system efficiency.
